# Is gross morphology of placenta, umbilical cord, and neonatal outcome in well-controlled gestational diabetes mellitus pregnancy different? A case-control study

**DOI:** 10.18502/ijrm.v13i6.7282

**Published:** 2020-06-30

**Authors:** Parichehr Pooransari, Atefeh Ebrahimi, Nataliya Nazemi, Fariba Yaminifar, Zhila Abediasl

**Affiliations:** ^1^Shohada Tajrish Hospital, Shahid Beheshti University of Medical Sciences, Tehran, Iran.; ^2^IVF Department, Bahman Hospital, Tehran, Iran.

**Keywords:** Placenta, Umbilical cord, Gestational diabetes mellitus.

## Abstract

**Background:**

The relation of placental gross morphology and the outcome of pregnancies complicated with diabetes mellitus in comparison with healthy pregnancies is not known. Identifying significant differences in pregnancy outcomes in Gestational Diabetes Mellitus (GDM) and healthy pregnancies by the means of morphologic measurements can induce the use of antenatal ultrasonography of placental parameters to predict pregnancy outcomes.

**Objective:**

This study aimed to evaluate the relationship between placental morphological parameters of the placenta and cord and the outcomes of pregnancies complicated with diabetes mellitus.

**Materials and Methods:**

In this case-control study, which was conducted at two referral perinatology center in Tehran between March 2017 and November 2018, 60 pregnant women with GDM who were controlled with either diet or insulin as the case group and 60 pregnant women without GDM as the control group were enrolled. The study population were selected from patients who had their prenatal care and delivery in Mahdieh and Shohadaye Tajrish Hospital. The data was collected by taking sickness history, using data from patients files, and measuring of placental and newborn parameters after delivery. GDM was diagnosed either by 75 gr or 100 gr oral glucose tolerance tests. Placenta parameters, umbilical cord features, and newborn outcomes were compared between the two groups.

**Results:**

Placental weight, diameter, number of lobes, thickness, placental weight to-newborn weight ratio, place of umbilical cord insertion, length, coiling, and diameter of the umbilical cord are similar in two groups. Newborn weight, NICU admission, ABG, and Apgar score are also the same in well-controlled GDM pregnancy and pregnancy without GDM.

**Conclusion:**

Good controlled GDM causes no difference in placental gross morphology and pregnancy outcome compared to a healthy pregnancy.

## 1. Introduction

Gestational diabetes mellitus (GDM) is one of the most common complications in pregnancy. It has been estimated that 7% of pregnancies in the US are complicated with diabetes melitus (DM) (1). GDM is a known risk factor for pregnancy complications such as macrosomia, shoulder dystocia, birth trauma, increased rate of cesarean delivery, and neonatal metabolic disorders. Long-term health side effects among children born to mother who has GDM include obesity, impairment of glucose tolerance. Women with history of GDM more often have subsequent diabetes (2). Development of placenta in diabetes has structural and functional differences. It depends on a glycemic control during placental development, type of treatment, and period of time when diabetes was not controlled (3). The placenta is a morpho-functional structure with a main metabolic role during pregnancy. It is the crucial organ responsible for nutrient uptake, waste elimination, and gas exchange between fetus and mother (4). During pregnancy associated with diabetes, the placenta undergoes some functional and structural pathologic changes, such as increased placental weight and placental lesions (5). Due to the growth-promoting and growth-restricting factors, DM complicates pregnancy and alters normal growth patterns of fetus and placenta (6).

Considering the possibility of the relation between the placenta morphology, umbilical cord, and newborn parameters with diabetes, this study was aimed to evaluate the relationship between placental morphological parameters such as weight, diameter, number of lobes, thickness, umbilical cord insertion, length, coiling and diameter of the umbilical cord with the outcome of pregnancies complicated with DM.

## 2. Materials and Methods

In this case-control study, 60 pregnant women with GDM who were controlled with either diet or insulin as the case group, and 60 pregnant women without GDM as the control group were enrolled. The control group were selected from pregnant women, referred to Mahdieh Hospital and Shohadaye Tajrish Hospital, Tehran, Iran between March 2017 and November 2018.

Our inclusion criteria for selecting the case group were gestational age ≥ 37 weeks at delivery and diagnosed gestational diabetes mellitus.

All women with delivery at < 37 weeks, pregnant women with other maternal medical diseases (PIH, chronic hypertension, DM type I and II started before pregnancy, other systemic diseases), multiple pregnancy, tumors of placenta (angioma etc.), two vessels umbilical cord were excluded from the study.

GDM was diagnosed either by 75 gr or by 100 gr oral glucose tolerance test.

Placenta parameters, umbilical cord features, and newborn outcomes were compared between the two groups.

The aim of glycemic control was HbA1C < %6, without significant hypoglycemia, Fasting blood sugar < 95 mg/dL and either, 1-hr postprandial < 140 mg/dL or 2-hr postprandial < 120 mg/dL according to American diabetes association (7). The control group had normal response to oral glucose tolerance test.

Data including maternal parameters (age, Body mass index (BMI), gestational age, parity, gravity), placental morphological parameters (after they were tagged and washed thoroughly to remove blood and mucus) such as weight, diameter from the widest part, number of lobes, thickness (that was taken from cord insertion area), umbilical cord insertion, length from placental end to the fetal end, coiling, diameter of umbilical cord in transverse cut, and newborn parameters (NICU admission, weight of newborn, ABG, Apgar score, presence of meconium in amniotic fluid) were collected.

Umbilical cord was considered vellamentous if it was inserted in the membranes before reaching the chorionic plate. Umbilical cord was considered marginal when ≤ 1 cm is left to placental margin, central when cord insertion place was ≤ 1 cm away from center, other types of cord insertion we named paracentral. The coiling index is the amount of coils divided by length of umbilical cord in cm. Coiling index was considered hypocoiled if it was below 0.1 coils/cm and was considered hypercoiled if it was above 0.3 coils/cm (8).

### Ethical consideration

Signed written informed consent forms were collected from the volunteers participating in the study. The ethics committee of Shahid Beheshti University of Medical Sciences, Tehran, Iran approved the study in order not to undermine the patient's rights (Code: IR.SBMU.MSP.REC.1396.476).

### Statistical analysis

Data were entered into SPSS-21 software. Data was assessed with Kolmogorov-Smimov test at first. Thereafter, data were analyzed using Chi-square and paired sample *t* tests. To examine the correlation between the variables, Pearson and Spearman correlation methods were used. Also confidence interval was 95% and the statistical significance was considered as p < 0.05.

## 3. Results

A total of 120 pregnant women participated in this case-control study (n = 60/each). All of the participants delivered live newborn at gestational age > 37 wk. Participants' demographic characteristics were showed in table I. The mean age of women in the case group was 30 ± 6.2 yr, while the mean age of those in the control group was 27.7 ± 6.2 yr. BMI, age, parity, and gravity in the case and control group didn't have significant differences (Table I).

Thirty-eight women in the case group and twenty-two women in the control group underwent cesarean delivery, the difference between the two groups was significant (p = 0.03).

Blood sugar in all mothers with GDM were well controlled; 55 women were controlled only by diet and 5 mothers consumed insulin.

The placental and umbilical cord gross morphology including placental weight, diameter, number of lobes, thickness, place of umbilical cord insertion, cord length, coiling, the diameter of umbilical cord, and placental weight-to-newborn weight ratio had no significant differences between the two groups (Table II).

The mean newborn weight in case group was 3380.8 ± 404.1 gr and in control group 3320 ± 432.1 gr (p = 0.427, with no significant differences). All newborn in case and control groups had Apgar score of 10 at the 5${}^{\mathrm{th}}$ min and normal ABG parameters. About 10% of the newborns in the case group and 8.3% newborns in the control group were hospitalized to NICU (p = 0.67). Four newborns (6.7%) in the control group and none in the case group had meconium excretion in amniotic fluid (p = 0.042, significant).

**Table 1:** Demographic characteristics of study participants in two groups


	**Control group**	**Case group**	**P-value**
Mean age (yr)*		27.7 ± 6.2	30 ± 6.2	0.331
Type of delivery**				
	**Cesarean**	22 (36.7)	38 (63.3)	
	**NVD**	38 (63.3)	22 (36.7)	0.003
BMI**				
	**< 25**	28 (46.7)	20 (33.3)	
	**25-29**	24 (44.0)	29 (48.3)	
	**> 30**	8 (13.3)	11 (18.3)	0.32
Gravity**				
	**1**	19 (31.7)	22 (36.7)		
	**> 1**	41 (68.3)	38 (63.3)	0.56
Parity**				
	**1**	23 (38.3)	31 (51.7)	
	**> 1**	38 (61.7)	29 (48.3)	0.14
Gestational age**				
	**37-37 + 6**	6 (10.0)	9 (15.0)	
	**38-38 + 6**	24 (40.0)	23 (38.3)	
	**39-39 + 6**	15 (25.0)	15 (25.0)	
	**> 40**	15 (25.0)	13 (21.7)	0.86
Type of diabetes control**				
	**Under diet**	0	55 (91.7)		
	**Under insulin**	0	5 (8.3)		
	**Without diet**	60 (100)	0	< 0.001
* Data presented as Mean ± SD; ** Data presented as n (%); Chi-square test NVD: Normal vaginal delivery; BMI: Body mass index				

**Table 2:** Parameters of placental and umbilical cord gross morphology in cases with GDM and control without GDM


	**Control group**	**Case group**	**P-value**
Placental weight${}^{*}$				
	**< 400**	2 (3.3)	0 (0.0)	
	**400-600**	28 (46.7)	27 (45.0)	
	**600-800**	22 (36.7)	30 (50.0)	
	**800-1000**	8 (13.3)	3 (5.0)	0.37${}^{a}$
Place of umbilical cord insertion*				
	**Central**	12 (20.0)	18 (30.0)		
	**Paracentral**	41 (68.3)	30 (50.0)		
	**Marginal**	5 (8.3)	12 (20.0)	
	**Velamentous**	2 (3.3)	0 (0.0)	0.037${}^{a}$
Number of placental lobes*				
	**One lobe**	52 (86.7)	56 (93.3)		
	**More than one lobe**	8 (13.3)	4 (6.7)		0.22${}^{a}$
Placental shape*					
	**Round**	28 (46.7)	25 (41.7)	
	**Oval**	32 (53.3)	34 (56.7)	
	**Irregular**	0 (0.0)	1 (1.7)	0.54${}^{a}$
	**Control group**	**Case group**	**P-value**	
Coiling*				
	**Hypocoiled**	12 (20)	9 (15.0)	
	**Normocoiled**	48 (80)	51 (85.0)	0.86${}^{a}$
Direction of coiling*				
	**Left**	50 (83.3)	50 (83.3)	
	**Right**	10 (16.8)	10 (16.7)	1${}^{a}$
Diameter of umbilical cord*				
	**2-Jan**	35 (58.3)	32 (53.3)	
	**> 2**	25 (41.7)	28 (46.7)	0.58${}^{a}$
Central thickness of placenta*				
	**< 2**	20 (33.3)	18 (30.0)	
	**4-Feb**	40 (66.7)	42 (70.0)	0.695${}^{a}$
Diameter of placenta*				
	**< 16**	15 (25)	15 (25.0)	
	**16-18.9**	20 (33.3)	18 (30.0)	
	**19-22**	21 (35)	24 (40)	
	**> 22**	4 (6.7)	3 (5.0)	0.93${}^{a}$
	Newborn weight (gr)**	3320 ± 432.1	3380.8 ± 404.1		0.50${}^{b}$
	Umbilical length (cm)**	52.2 ± 9.7	50.95 ± 10.2		0.75${}^{b}$
Placental weight to newborn*				
	**< 1:6**	42 (70)	45 (75.0)	
	**1:6-1:7**	14 (23.3)	12 (20.0)	
	**> 1:7**	4 (6.7) weight ratio	3 (5.0)	0.819${}^{a}$
* Data presented as Mean±SD. ** Data presented as n (%).${}^{a}$Chi-square, ${}^{b}$ *t* test				

## 4. Discussion

This investigation assessed the differences between placental gross morphology and pregnancy outcome in GDM and healthy pregnancy. The study suggests that there is no difference in the aforementioned parameters between a healthy pregnancy and a good controlled GDM pregnancy. Good controlled GDM group and healthy pregnancy had almost same parameters of placental gross morphology and pregnancy outcome. This shows the effect of well GDM treatment.

So, ultrasound investigation of the placenta and umbilical cord antepartum in pregnancy with well-controlled GDM will not help to predict bad pregnancy outcome in these patients. In our research four newborns in the control group had meconium in amniotic fluid but none in the case group had meconium excreted in the amniotic fluid. These results might be due to immaturity of the gastrointestinal tract in newborns of mothers with GDM. But further evaluation is required because all four cases in the control group were born by normal vaginal delivery, and more cesarean section was performed in the GDM group; this can cause differences because in elective cesarean section we rarely see meconium in amniotic fluid compared to normal vaginal delivery.

It is found that umbilical coiling index (UCI) can be a marker of adverse pregnancy outcomes, for example; hypocoiling (< 0.12) is associated with placental abruption, decreased amniotic fluid, preeclampsia, abnormal fetal heart rate.

Hypercoiling (> 0.36) has relation with increased amniotic fluid, congenital disorders, delivery by cesarean section, and respiratory distress of the newborn (9). This finding is in some ways consistent with a study conducted in 2019 which concluded that GDM is connected to increase of hospital admission, congenital abnormalities, emergent delivery by cesarean section, PROM, preterm birth. Increase in the UCI is connected to macrosomia and meconium-stained amniotic fluid in patients with GDM (10). Further, in some investigations, the evaluations showed that antenatal UCI that was performed by ultrasound at 18-23 wk of gestation could predict postnatal UCI with strong diagnostic accuracy in GDM group (11, 12).

In a study assessing the possible adverse effects of uncontrolled DM on morphometric of the umbilical cord and its vessels, the investigation showed that single umbilical artery (SUA) was much more frequently seen in mothers with gestational diabetes compared to normal pregnancy. It was shown that lean cord and SUA were connected to GDM and has association with unfavorable fetal outcome (13). Our study shows that proper glycemic control has managed these adverse effects in our patients with GDM.

It was shown in another investigation that the circumference and the mean diameter of umbilical cord is larger in GDM mothers than non-diabetics (p = 0.0001). But difference in type of insertion, coiling index, false knots, and length of umbilical cord were insignificant between GDM and healthy pregnant women.

Also, the number of umbilical cord vessels in both groups was the same and true knots were absent (14).

Contrary to the results of our study in the study by Rabia Arshad and coworkers the group of GDM on diet had heavier placenta compared to the control's. They also demonstrated that villous immaturity, chorangiosis, infarction, and syncytial knots in light microscopy were present in the GDM group versus the control group (15).

### Limitation

The limitations to this study are that all patients of case group had well-controlled GDM, so, we suggest studying poor controlled GDM next time and comparing it with healthy pregnancies. Most women in GDM group had elective cesarean sections, but in control group the mothers delivered through normal vaginal delivery NVD. Although it can't change placental morphological parameters, it may influence the pregnancy outcome. Most women in this study controlled their GDM by diet, it is recommended to study mothers controlling GDM with insulin and compare the results.

## 5. Conclusion

This study identifies that good controlled GDM at pregnancy makes the outcome of the pregnancy and gross structure of placenta equal to healthy pregnancies without GDM. It shows the significant role of prenatal care and early GDM diagnosis and treatment in newborns' health.

##  Conflict of Interest

There is no conflict of interest to be declared.
